# Pharmacokinetics, Safety, and Clinical Efficacy of Cannabidiol Treatment in Osteoarthritic Dogs

**DOI:** 10.3389/fvets.2018.00165

**Published:** 2018-07-23

**Authors:** Lauri-Jo Gamble, Jordyn M. Boesch, Christopher W. Frye, Wayne S. Schwark, Sabine Mann, Lisa Wolfe, Holly Brown, Erin S. Berthelsen, Joseph J. Wakshlag

**Affiliations:** ^1^Department of Clinical Sciences, College of Veterinary Medicine, Cornell University, Ithaca, NY, United States; ^2^Department of Molecular Medicine, College of Veterinary Medicine, Cornell University, Ithaca, NY, United States; ^3^Department of Population Medicine, College of Veterinary Medicine, Cornell University, Ithaca, NY, United States; ^4^Proteomic and Metabolomic Facility, Colorado State University, Fort Collins, CO, United States; ^5^Metzger Animal Hospital, State College, PA, United States

**Keywords:** cannabidiol, CBD oil, hemp, canine, osteoarthritis, pharmacokinetic

## Abstract

**Objectives:** The objectives of this study were to determine basic oral pharmacokinetics, and assess safety and analgesic efficacy of a cannabidiol (CBD) based oil in dogs with osteoarthritis (OA).

**Methods:** Single-dose pharmacokinetics was performed using two different doses of CBD enriched (2 and 8 mg/kg) oil. Thereafter, a randomized placebo-controlled, veterinarian, and owner blinded, cross-over study was conducted. Dogs received each of two treatments: CBD oil (2 mg/kg) or placebo oil every 12 h. Each treatment lasted for 4 weeks with a 2-week washout period. Baseline veterinary assessment and owner questionnaires were completed before initiating each treatment and at weeks 2 and 4. Hematology, serum chemistry and physical examinations were performed at each visit. A mixed model analysis, analyzing the change from enrollment baseline for all other time points was utilized for all variables of interest, with a *p* ≤ 0.05 defined as significant.

**Results:** Pharmacokinetics revealed an elimination half-life of 4.2 h at both doses and no observable side effects. Clinically, canine brief pain inventory and Hudson activity scores showed a significant decrease in pain and increase in activity (*p* < 0.01) with CBD oil. Veterinary assessment showed decreased pain during CBD treatment (*p* < 0.02). No side effects were reported by owners, however, serum chemistry showed an increase in alkaline phosphatase during CBD treatment (*p* < 0.01).

**Clinical significance:** This pharmacokinetic and clinical study suggests that 2 mg/kg of CBD twice daily can help increase comfort and activity in dogs with OA.

## Introduction

Routine nonsteroidal anti-inflammatory drug (NSAID) treatments, though efficacious, may not provide adequate relief of pain due to osteoarthritis (OA) and might have potential side effects that preclude its use, particularly in geriatric patients with certain comorbidities, such as kidney or gastrointestinal pathologies (1–4). In a systematic review of 35 canine models of OA and 29 clinical trials in dogs, treatment with NSAIDs caused adverse effects in 35 of the 64 (55%) studies, most commonly being gastro-intestinal signs (3). Although other pharmacological agents are advocated, such as gabapentin or amantadine, there is little evidence regarding their efficacy in dogs with chronic or neuropathic pain related to OA. Recent medical interest in alternative therapies and modalities for pain relief has led many pet owners to seek hemp related products rich in cannabinoids.

The endocannabinoid receptor system is known to play a role in pain modulation and attenuation of inflammation (5–7). Cannabinoid receptors (CB1 and CB2) are widely distributed throughout the central and peripheral nervous system (8–10) and are also present in the synovium (11). However, the psychotropic effects of certain cannabinoids prevent extensive research into their use as single agents for pain relief (5, 12). The cannabinoids are a group of as many as 60 different compounds that may or may not act at CB receptors. One class of cannabinoids, cannabidiol (CBD), may actually be an allosteric non-competitive antagonist of CB receptors (13). In lower vertebrates, CBD is also reported to have immunomodulatory (14), anti-hyperalgesic (15, 16), antinociceptive (17, 18), and anti-inflammatory actions (5, 19), making it an attractive therapeutic option in dogs with OA. Currently there are several companies distributing nutraceutical derivatives of industrial hemp, rich in cannabinoids for pets, yet little scientific evidence regarding safe and effective oral dosing exists.

The objectives of this study were to determine: (1) single-dose oral pharmacokinetics, (2) short-term safety, and (3) efficacy of this novel CBD-rich extract, as compared to placebo, in alleviating pain in dogs with OA. Our underlying hypotheses were that appropriate dosing of CBD-rich oil would safely diminish perceived pain and increase activity in dogs with OA.

## Materials and methods

### CBD oil and protocols approval

The industrial hemp used in this study was a proprietary hemp strain utilizing ethanol and heat extraction with the final desiccated product reconstituted into an olive oil base containing ~10 mg/mL of CBD as an equal mix of CBD and carboxylic acid of CBD (CBDa), 0.24 mg/mL tetrahydrocannabinol (THC), 0.27 mg/mL cannabichromene (CBC), and 0.11 mg/mL cannabigerol (CBG); all other cannabinoids were less than 0.01 mg/mL. Analysis of five different production runs using a commercial analytical laboratory (MCR Laboratories, Framingham, MA) show less than a 9% difference across batches for each of the detected cannabinoids listed above. The study was performed after the Cornell University institutional animal care and use committee (IACUC) approved the study following the guidelines for animal use according to the IACUC. Client owned dogs were enrolled after informed consent in accordance with the Declaration of Helsinki.

### Pharmacokinetics

An initial investigation into single-dose oral pharmacokinetics was performed with 4 beagles (3.5–7 years, male castrated, 10.7–11.9 kg). Each dog received a 2 mg/kg and an 8 mg/kg oral dosage of CBD oil, with a 2-week washout period between each experiment. The dogs were fed 2 h after dosing. Physical examination was performed at 0, 4, 8, and 24 h after dosing. Attitude, behavior, proprioception, and gait were subjectively evaluated at each time point during free running/walking and navigation around standard traffic cones (weaving). Five milliliters of blood was collected at time 0, 0.5, 1, 2, 4, 8, 12, and 24 h after oil administration. Blood samples were obtained via jugular venipuncture and transferred to a coagulation tube for 20 min. Samples were centrifuged with a clinical centrifuge at 3,600 × g for 10 min; serum was removed and stored at −80°C until analysis using liquid chromatography-mass spectrometry (LC-MS) at Colorado State University Core Mass Spectrometry facility.

### Extraction of CBD from canine serum and mass spectrometry analysis

CBD was extracted from canine serum using a combination of protein precipitation and liquid-liquid extraction using n-hexane as previously described (20), with minor modifications for microflow ultra-high pressure liquid chromatography (UHPLC). Briefly, 0.05 mL of canine serum was subjected to protein precipitation in the presence of ice-cold acetonitrile (80% final concentration), spiked with deuterated CBD as the internal standard (0.06 mg/mL, CDB-d3 Cerilliant, Round Rock, TX, USA). 0.2 mL of water was added to each sample prior to the addition of 1.0 mL of hexane to enhance liquid-liquid phase separation. Hexane extract was removed and concentrated to dryness under laboratory nitrogen. Prior to LC-MS analysis, samples were resuspended in 0.06 mL of 100% acetonitrile. A standard curve using the CBD analytical standard was prepared in canine serum non-exposed to CBD and extracted as above. Cannabidiol concentration in serum was quantified using a chromatographically coupled triple-quadropole mass spectrometer (UHPLC-QQQ-MS) using similar methods as previously described (21).

### CDB serum concentration data analysis

From the UHPLC-QQQ-MS data, peak areas were extracted for CBD detected in biological samples and normalized to the peak area of the internal standard CBD-d3, in each sample using Skyline (22) as well as an in-house R Script (www.r-project.org). CBD concentrations were calculated to nanograms per mL of serum as determined by the line of regression of the standard curve (*r*^2^ = 0.9994, 0–1,000 ng/mL). For this assay, the limits of detection (LOD) and limits of quantification (LOQ) represent the lower limits of detection and quantification for each compound in the matrix of this study (23, 24). Pharmacokinetic variables were estimated by means of non-compartmental analysis, utilizing a pharmacokinetic software package (PK Solution, version 2.0, Montrose, CO, USA).

### Inclusion and exclusion criteria for the clinical trial

The study population consisted of client-owned dogs presenting to Cornell University Hospital for Animals for evaluation and treatment of a lameness due to OA. Dogs were considered for inclusion in the study if they had radiographic evidence of OA, signs of pain according to assessment by their owners, detectable lameness on visual gait assessment and painful joint(s) on palpation. Each dog had an initial complete blood count ([CBC] Bayer Advia 120, Siemens Corp., New York, NY, USA) and serum chemistry analysis (Hitachi 911, Roche Diagnostics, Indianapolis, IN, USA) performed to rule out any underlying disease that might preclude enrolment. Elevations in alkaline phosphatase (ALP), alanine aminotransferase (ALT), and aspartate aminotransferase (AST) were allowed if prior hepatic ultrasound was deemed within normal limits except for potential non-progressive nodules (possible hepatic nodular hyperplasia). All owners completed a brief questionnaire to define the affected limb(s), duration of lameness, and duration of analgesic or other medications taken. All dogs underwent radiographic examination of affected joints and a radiologist confirmed the presence or absence of OA, and excluded the presence of concomitant disease that might preclude them from enrolment (i.e., lytic lesions).

During the trial, dogs were only allowed to receive NSAIDs, fish oil, and/or glucosamine/chondroitin sulfate without any change in these medications for 4 weeks prior to or during the 10-week study period as standard of care for the disease process. Other analgesic medications used, such as gabapentin and tramadol, were discontinued at least 2 weeks prior to enrolment. Dogs were excluded if they had evidence of renal, uncontrolled endocrine, neurologic, or neoplastic disease, or were undergoing physical therapy. Every dog was fed its regular diet with no change allowed during the trial.

### Clinical trial

The study was a randomized, placebo-controlled, owner and veterinarian double-blind, cross-over trial. Dogs received each of two treatments in random order (Randomizer iPhone Application): CBD, 2 mg/kg every 12 h, or placebo (an equivalent volume of olive oil with 10 parts per thousands of anise oil and 5 parts per thousands of peppermint oil to provide a similar herbal smell) every 12 h. Each treatment was administered for 4 weeks with a 2-week washout period in between treatments. Blood was collected to repeat complete blood counts and chemistry analysis at weeks 2 and 4 for each treatment.

At each visit, each dog was evaluated by a veterinarian based on a scoring system previously reported (25) as well as by its owner (canine brief pain inventory [CBPI], Hudson activity scale) before treatment initiation and at weeks 2 and 4 thereafter (26–28).

### Statistical analysis

Initial power analysis was performed to assess number of dogs needed for this study as a cross over design with a power set 0.80 and alpha of 0.05 using prior data suggesting a baseline CBPI or Hudson score change of ~15 points (two tailed) with a standard deviation of 20. When calculated it was assumed that 14 dogs would be necessary to find differences in outcomes of interest (29).

Statistical analysis was performed with a commercially available software package (JMP 12.0, Cary, NC, USA). All continuous data were assessed utilizing a Shapiro–Wilk test for normality. Considering a majority of our blood, serum and scoring data were normally distributed a mixed model analysis was used to analyze these outcomes, including the fixed effects of treatment, time, sequence of treatment assignment, gender, age, NSAID usage, treatment × time; as well as random effects of observation period, period nested within dog, time point nested within period nested within dog to account for the hierarchical nature of data in a cross-over design as well as repeated measurements for each dog. For ordinal veterinary scoring data a similar linear mixed model was used, but differences from baseline were first calculated to approximate a normal distribution to meet assumptions for a mixed model analysis. Residual diagnostics of all final models showed that residuals were normally distributed and fulfilled the assumption of homoscedasticity, and assumptions where therefore met. This statistical modeling approach allowed for adequate control of hierarchical data structure necessary in a cross-over design, as well as for the performance of easily interpretable time × treatment Tukey *post-hoc* comparisons that were our main interest, as compared to an ordinal logistical regression (30, 31). To control for baseline differences and therefore the possible difference in relative change in CBPI pain, and activity interference assessments and Hudson scoring across dogs, the initial CPBI or Hudson Scores were included for these analyses as a covariate. Pairwise comparisons between all-time points of both groups were corrected for multiple comparisons with Tukey's *post-hoc* tests to examine the interaction of time and treatment variables, and to assess differences between change from baseline at any time point as they related to treatment. A *p*-value of less than 0.05 was defined as the significance cut-off.

## Results

### Pharmacokinetics

Pharmacokinetics demonstrated that CBD half-life of elimination median was 4.2 h (3.8–6.8 h) for the 2 mg/kg dose, and 4.2 h (3.8–4.8 h) for the 8 mg/kg dose (Table 1). Median maximal concentration of CBD oil was 102.3 ng/mL (60.7–132.0 ng/mL; 180 nM) and 590.8 ng/mL (389.5–904.5 ng/mL; 1.2 uM) and was reached after 1.5 and 2 h, respectively, for 2 and 8 mg/kg doses. No obvious psychoactive properties were observed on evaluation at any time point during the 2 and 8 mg/kg doses over 24 h. These results led to dosing during the clinical trial at 2 mg/kg body weight every 12 h, due the cost prohibitive nature of 8 mg/kg dosing for most larger patients, the impractical nature of more frequent dosing, the volume of oil necessary and anecdotal reports surrounding 0.5-2 mg/kg dosing recommended by other vendors.

**Table 1:** Serum pharmacokinetic of single oral dosing (2 mg and 8 mg/kg) of CBD oil in dogs.

	**Cmax (ng/mL)**	**Tmax (h)**	**T1/2 elim (h)**	**AUC 0-t (ng-hr/mL)**	**MRT (h)**
**DOSE (2 mg/kg)**					
Dog 1	61	1	4.4	183	6.0
Dog 2	132	1	3.9	351	4.2
Dog 3	102	2	3.8	382	5.1
Dog 4	101	2	6.8	437	9.1
Median (Range)	102 (61–132.0)	1.5 (1.0–2.0)	4.2 (3.8–6.8)	367 (183–437)	5.6 (4.2–9.1)
**DOSE (8 mg/kg)**					
Dog 1	499	2	3.8	2,928	5.7
Dog 2	389	1	4.8	1,753	7.0
Dog 3	905	2	4.2	3,048	5.1
Dog 4	682	2	4.1	2,389	5.2
Median (Range)	591 (389–905)	2.0 (1.0–2.0)	4.2 (3.8–4.8)	2,658 (1,753–3,048)	5.6 (5.1–7.0)

*Cmax, maximum concentration; Tmax, time of maximum concentration; T1/2 el, half-life of elimination; AUC 0-t, area under the curve (time 0–24 h); MRT, median residence time*.

### Dogs included in the clinical trial

Twenty-two client-owned dogs with clinically and radiographically confirmed evidence of osteoarthritis were recruited. Sixteen of these dogs completed the trial and were included in the analyses; their breed, weight, age, sex, worse affected limb, radiographic findings, use of NSAIDs and sequence of treatments are summarized in Table 2. Dogs were removed due to osteosarcoma at the time of enrolment, gastric torsion (placebo oil), prior aggression issues (CBD oil), pyelonephritis/kidney insufficiency (CBD oil), recurrent pododermatitis (placebo oil), and diarrhea (placebo oil).

**Table 2:** Characteristics of dogs enrolled in a placebo-controlled study investigating the effects of CBD on osteoarthritis.

**Breed**	**Weight (kg)**	**Age (years)**	**Sex**	**Radiographic findings and OA localization**	**NSAID**
Rottweiler	35.3	10	FS	- Moderate, intracapsular swelling with moderate osteophytosis, left stifle	Carprofen (2.1 mg/kg BID)
Mix	30.6	13	MC	- Moderate-to-severe, right-shoulder osteoarthrosis; mild, left-shoulder osteoarthrosis - Moderate-to-severe, bilateral hip osteoarthrosis	None
Mix	27.2	9	FS	- Moderate medial coronoid remodeling (with fragmentation on the right) and bilateral elbow osteoarthrosis	None
Mix	30.5	14	MC	- Moderate enthesiopathies on right carpus; mild, left-antebrachiocarpal osteoarthrosis - Bilateral moderate coxofemoral osteoarthrosis	None
Mix	23.5	10	FS	- Moderate bilateral stifle osteoarthrosis and moderate intracapsular swelling	Carprofen (2.2 mg/kg)
Mix	28.1	10	FS	- Moderate bilateral elbow osteoarthrosis - Moderate left-stifle osteoarthrosis with intracapsular swelling	Metacam (0.1 mg/kg
English Bulldog	25.2	8	MC	- Severe osteoarthrosis, left elbow - Moderate intracapsular swelling and mild osteoarthrosis, right stifle	Carprofen (2 mg/kg BID)
German Shorthaired Pointer	21.5	14	FS	- Moderate bilateral elbow osteoarthrosis	Carprofen (2.4 mg/kg BID)
Labrador Retriever	26.1	13	FS	- Bilateral severe stifle osteoarthrosis due to cranial cruciate ligament disease	Meloxicam (0.1 mg/kg SID)
Mix	18.2	13	FS	- Bilateral moderate elbow osteoarthrosis and medial epicondylitis	Meloxicam (0.1 mg/kg SID)
Mix	22	9	FS	- Moderate, stifle osteoarthrosis with moderate intracapsular swelling	None
Bernese Mountain Dog	50	3	M	- Bilateral severe elbow osteoarthritis, medial coronoid disease, and medial epicondylitis	Carprofen (2 mg/kg SID)
Belgian Malinois	25.1	9	FS	- Severe bilateral elbow osteoarthrosis - Bilateral moderate hip osteoarthrosis	Carprofen (2 mg/kg BID)
Mix	28.6	13	FS	- Severe bilateral elbow osteoarthritis - Severe bilateral hip osteoarthritis	None
Border Collie	22	14	MC	- Severe thoracolumbosacral osteophytosis - Multifocal carpal enthesiophytes	None
Beagle	17.6	5	MC	- Mild left elbow osteoarthrosis, with possible medial coronoid disease - Moderate-to-severe bilateral stifle osteoarthrosis	None

*FS, female spayed; MC, male castrated; Mix, mixed breed; SID, once daily; BID, twice daily*.

### Clinical trial

CBPI and Hudson change from baseline scores showed a significant decrease in pain and increase in activity (*p* < 0.01) at week 2 and 4 during CBD treatment when compared to baseline week 0, while no other statistical significances were observed across treatment in this cross-over design (Table 3). Lameness as assessed by veterinarians showed an increase from baseline in lameness with age (*p* < 0.01), whereas NSAID use (*p* = 0.03) reduced lameness scores. Veterinary pain scores showed a decrease from baseline in dogs on NSAIDs (*p* < 0.01). CBD oil resulted in a decrease in pain scores when compared to baseline on evaluation at both week 2 and week 4 (*p* < 0.01 and *p* = 0.02, respectively), and week 2 CBD oil treatment was lower than baseline placebo treatment (*p* = 0.02) and week 4 placebo treatment (*p* = 0.02). No other veterinary pain comparisons were statistically significant. No changes were observed in weight-bearing capacity when evaluated utilizing the veterinary lameness and pain scoring system (Table 3).

**Table 3:** Canine Brief Pain Inventory (Pain and Activity questions) and Hudson Scale mean and standard deviation; lameness, weight-bearing and pain scores median and ranges at each time for cannabidiol (CBD) and placebo oils.

	**CBD oil**			**Placebo oil**		
	**Week 0**	**Week 2**	**Week 4**	**Week 0**	**Week 2**	**Week 4**
CBPI Pain (0–40)	21 ± 8	14 ± 6^*^	14 ± 8^*^	17 ± 7	19 ± 9	19 ± 9
CBPI activity interference (0–60)	35 ± 15	25 ± 15^*^	26 ± 14^*^	27 ± 15	29 ± 15	31 ± 16
Hudson (0–110)	54 ± 13	67 ± 15^*^	67 ± 10^*^	65 ± 14	64 ± 16	60 ± 19
Veterinary lameness§	3 (1–4)	3 (1–4)	3 (1–4)	3 (2–4)	3 (2–4)	3 (1–4)
Veterinary pain ∫	3 (3–4)	3 (2–4)^*^	3 (1–4)^*^	3 (2–4)^**^	3 (2–4)	3 (2–4)^**^
Veterinary weight-bearing =	2 (1–3)	2 (1–3)	2 (1–3)	2 (1–3)	2 (1–3)	2 (1–3)

* *Represents significant difference (p < 0.05) from baseline week 0 of CBD treatment*.

** Represents significant differences (p < 0.05) from week 2 of CBD oil treatment. §Lameness was scored as follows: 1 = no lameness observed/walks normally, 2 = slightly lame when walking, 3 = moderately lame when walking, 4 = severely lame when walking, 5 = reluctant to rise and will not walk more than 5 paces. ∫Pain on palpation was scored as follows: 1 = none, 2 = mild signs, dog turns head in recognition, 3 = moderate signs, dog pulls limb away, 4 = severe signs, dog vocalizes or becomes aggressive, 5 = dog will not allow palpation. = Weight-bearing was scored as follows: 1 = equal on all limbs standing and walking, 2 = normal standing, favors affected limb when walking, 3 = partial weight-bearing standing and walking, 4 = partial weight-bearing standing, non-weight-bearing walking, 5 = non-weight-bearing standing and walking.

**Figure 1:**
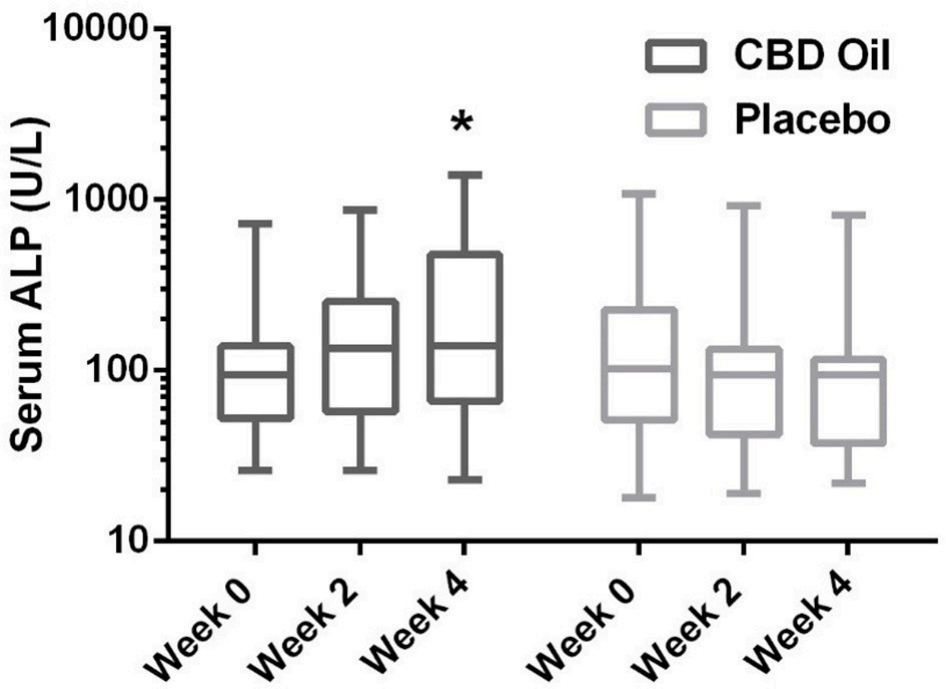
Box-and-whisker plot of serum alkaline phosphatase (ALP) activity at each time for treatment and placebo oils. Box represents the mean and 25th and 75th percentile and the whiskers represent the 99th and 1st percentiles. ^*^Indicates a significant difference (*p* < 0.05) from week 0 CBD treatment.

Chemistry analysis and CBC were performed at each visit. No significant change in the measured CBC values was noted in either the CBD oil or placebo treated dogs (data not shown). Serum chemistry values were not different between placebo compared to CBD oil (Table 4), except for alkaline phosphatase (ALP) which significantly increased over time from baseline by week 4 of CBD oil treatment (p < 0.01); with nine of the 16 dogs showing increases over time (Figure 1). Glucose was increased in dogs receiving the placebo oil at each time point (p = 0.04) and creatinine levels increased over time in both dogs receiving CBD oil and those receiving placebo oil (p < 0.01); though all values remained within reference ranges. Other notable significances in serum chemistry values were associated with primarily age or NSAID use. An increase in age was associated with significantly higher blood urea nitrogen (BUN; p < 0.01), calcium (p = 0.01), phosphorus (p < 0.01), alanine aminotransferase (ALT; p = 0.03), alkaline phosphatase (ALP; p = 0.01), gamma glutamyltransferase (GGT; p = 0.02), globulin (p = 0.02), and cholesterol (p < 0.01) values. NSAID use was associated with significantly higher BUN (p = 0.003), and creatinine (p = 0.017), and significant decreases in total protein (p < 0.001) and serum globulin (p < 0.001).

**Table 4:** Serum chemistry values of dogs receiving CBD or placebo oils.

	**Reference**	**CBD oil**			**Placebo oil**		
		**Week 0**	**Week 2**	**Week 4**	**Week 0**	**Week 2**	**Week 4**
Sodium	145–153 mEq/L	149 ± 3	149 ± 2	149 ± 1	149 ± 1	149 ± 2	149 ± 2
Potassium	4.1–5.6 mEq/L	4.9 ± 0.3	4.9 ± 0.5	4.9 ± 0.3	4.8 ± 0.4	4.9 ± 0.4	4.9 ± 0.3
Chloride	105–116 mEq/L	110 ± 3	109 ± 3	109 ± 2	110 ± 2	110 ± 2	110 ± 2
SUN	10–32 mg/dL	20 ± 9	20 ± 7	20 ± 6	19 ± 6	21 ± 7	19 ± 6
Creatinine	0.6–1.4 mg/dL	1.0 ± 0.3	1.1 ± 0.3^*^	1.0 ± 0.3^*^	0.9 ± 0.3	1.0 ± 0.3^*^	1.0 ± 0.3^*^
Calcium	9.3–11.4 mg/dL	10.4 ± 0.5	10.4 ± 0.4	10.3 ± 0.4	10.4 ± 0.6	10.4 ± 0.4	10.4 ± 0.4
Phosphorus	2.9–5.2 mg/dL	3.8 ± 0.8	3.9 ± 0.8	3.9 ± 0.6	4.0 ± 0.7	3.9 ± 0.6	4.0 ± 0.5
Magnesium	1.4–2.2 mg/dL	1.8 ± 0.2	1.8 ± 0.2	1.8 ± 0.2	1.8 ± 0.1	1.8 ± 0.1	1.8 ± 0.1
Glucose	63–118 mg/dL	92 ± 9	89 ± 9	92 ± 9	97 ± 10^*^	93 ± 8	97 ± 10^*^
ALT	20–98 U/L	93 ± 86	93 ± 88	114 ± 119	90 ± 89	222 ± 606	166 ± 284
AST	14–51 U/L	31 ± 8	33 ± 13	34 ± 16	30 ± 8	56 ± 99	45 ± 34
ALP	17–111 U/L	160 ± 212	238 ± 268	323 ± 407^*^	204 ± 287	186 ± 287	175 ± 248
GGT	0–6 U/L	4 ± 3	3 ± 2	3 ± 2	3 ± 2	4 ± 6	5 ± 4
Bilirubin	0.0–0.2 mg/dL	0.1 ± 0.1	0.0 ± 0.1	0.1 ± 0.1	0.0 ± 0.1	0.0 ± 0.1	0.0 ± 0.1
Total protein	5.3–7.0 g/dL	6.3 ± 0.4	6.4 ± 0.5	6.3 ± 0.4	6.3 ± 0.4	6.3 ± 0.4	6.3 ± 0.4
Albumin	3.1–4.2 g/dL	3.7 ± 0.2	3.7 ± 0.2	3.7 ± 0.2	3.7 ± 0.2	3.7 ± 0.2	3.7 ± 0.2
Globulin	1.9–3.6 g/dL	2.6 ± 0.3	2.6 ± 0.4	2.6 ± 0.4	2.6 ± 0.4	2.6 ± 0.4	2.6 ± 0.4
Cholesterol	138–332 mg/dL	291 ± 64	301 ± 62	302 ± 62	295 ± 71	300 ± 71	308 ± 83
CK	48–260 U/L	148 ± 81	147 ± 59	134 ± 61	139 ± 57	158 ± 80	168 ± 105

Data presented at mean + standard deviations. Asterisk

(*) *indicates significantly different (p < 0.05) serum concentration from baseline week 0 CBD treatment. SUN, serum urea nitrogen; ALT, alanine animotranferase; AST, aspartate animotransferase; ALP, alkaline phosphatase; GGT, gamma glutamyl transferase; CK, creatine kinase*.

## Discussion

To date, an objective evaluation of the pharmacokinetics of a commercially available industrial hemp product after oral dosing in dogs is absent. This study showed that the terminal half-life of oral CBD, as the most abundant cannabinoid in this specific preparation when in an oil base, was between 4 and 5 h, suggesting it was bioavailable with a dosing schedule of 2 mg/kg at least twice daily. This half-life was shorter than a previous report after intravenous (1.88–2.81 and 3.75–5.63 mg/kg) and oral (7.5–11.25 mg/kg) administration (32). In the intravenous study, CBD distribution was rapid, followed by prolonged elimination with a terminal half-life of 9 h. When examining prior oral CBD bioavailability it was determined to be low and highly variable (0–19% of dose) with three dogs showing no absorption. This may be due to the first pass effect in the liver, and the product was not in an oil base, but a powder within a gelatin capsule being a different delivery vehicle (32). After initially seeing no neurological effects at the 2 mg/kg dose a 8 mg/kg dose was chosen to assess the potential neurological effects since mistaken overdosing can occur clinically, and a higher dose might have been necessary since the prior study showed poor absorption. Although our dogs were fasted the delivery vehicle was olive oil which is a food item. The absorption may be greater and more consistent because of the oil-based vehicle which may be due to the lipophilic nature of CBD, hence delivery with food may be preferable (32, 33). As previously demonstrated, CBD biotransformation in dogs involves hydroxylation, carboxylation and conjugation, leading to relatively rapid elimination suggesting a more frequent dosing schedule (34). The dosing schedule of twice per day was chosen due to the practical nature of this dosing regimen even though the elimination is well within a three or four time a day dosing regimen. Our hope was that the lipophilic nature of CBD would allow for a steady state over time, and future studies examining 24 h pharmacokinetics with different dosing regimens with larger numbers of dogs, and steady state serum pharmacokinetics after extended treatment in a clinical population are sorely needed.

The main objective of this study was to perform an owner and veterinary double-blinded, placebo-controlled, cross-over study to determine the efficacy of CBD oil in dogs affected by OA. Despite our small sample size, short study duration and heterogeneity of OA signs, CBPI and Hudson scores showed that CBD oil increase comfort and activity in the home environment for dogs with OA. Additionally, veterinary assessments of pain were also favorable. Although a caregiver placebo effect should be considered with subjective evaluations by owners and veterinarians (35), the cross-over design limits confounding covariates since each dog serves as its own control. Our statistical model controlled for the possible effect of treatment sequence. The lack of a placebo effect in our study may be due to nine of the 16 owners being intimately involved in veterinary medical care, all of whom have an understanding of the placebo effect making them more cognizant of improvements when providing feedback. In addition, there was a noticeable decrease in Hudson scores and rise in CBPI scores during the initiation placebo treatment suggesting a potential carry over effect of CBD treatment indicating that a longer washout period might be indicated in future studies. This carry over effect may have resulted in some improved perceptions at the initiation of the placebo treatment which were eliminated by week 4 of placebo treatment, underscoring the importance of longer term steady state PK studies in dogs.

There was no significant difference in subjective veterinary lameness score and weight-bearing capacity throughout the study. Kinetic data was obtained from these dogs (data not shown), however 11 of the 16 dogs had significant bilateral disease (stifle, coxofemoral, or elbow) making evaluation of peak vertical force or symmetry tenuous at best. Unilateral disease in any of the aforementioned joints would be ideal to study the kinetic effects of this or similar extracts for pain relief leading to better objective outcomes. The population we used in our investigation was representative of dogs presenting in a clinical setting for management of OA and represents the typical OA patient.

Currently, NSAIDs are the primary treatment for OA and are associated with negative effects on the gastrointestinal tract and glomerular filtration (2). In the current study, no significant difference was noted in BUN, creatinine, or phosphorus between dogs treated with the CBD oil vs. the placebo oil, while NSAID treatment resulted in a higher creatinine concentration. A mild rise in creatinine from baseline was noted in both groups at weeks 2 and 4, the hydration status of the dogs was unknown; however changes in albumin sodium, and chloride were unchanged suggesting euhydration, and all creatinine values remained within the reference interval. Increased ALP activity is fairly sensitive for hepatobiliary changes in this age group, but not specific. Increased ALP activity noted in nine dogs in the CBD treatment group may be an effect of the hemp extract attributed to the induction of cytochrome p450 mediated oxidative metabolism of the liver (reported previously with prolonged exposure to cannabis) (36–38). Other causes of cholestasis, increased endogenous corticosteroid release from stress, or a progression of regenerative nodular hyperplasia of the liver cannot be ruled out. Without concurrent significant rise in ALT in the CBD treatment to support hepatocellular damage, or biopsy for further clarification, the significance is uncertain. As such, it may be prudent to monitor liver enzyme values (especially ALP) while dogs are receiving industrial hemp products until controlled long term safety studies are published.

A recent survey reported that pet owners endorse hemp based treats and products because of perceived improvement in numerous ailments, as hemp products were moderately to very helpful medicinally (39). Some of the conditions thought to be relieved by hemp consumption were: pain, inflammation, anxiety and phobia, digestive system issue, and pruritus (39). One immunohistochemical study suggested that cannabinoids could protect against the effects of immune-mediated and inflammatory allergic disorders in dogs (40) whereas another uncontrolled study suggested that CBD has anticonvulsant and anti-epileptic properties in dogs (41). The apparent analgesic effect of the industrial hemp based oil observed in the present study may be attributable to downregulation of cylooxygenase enzymes, glycine interneuron potentiation, transient receptor potential cation receptor subfamily V1 receptor agonism (peripheral nerves), and/or g-protein receptor 55 activation (immune cells), influencing nociceptive signaling and/or inflammation (14, 42, 43).

The industrial hemp product used in this study is a proprietary strain-specific extract of the cannabinoids outlined in the methods with relatively high concentrations of CBD and lesser quantities of other cannabinoids as well as small amounts of terpenes that may have synergistic effects often termed the “entourage effect.” This brings to light that fact that different strains of cannabis produce differing amounts of CBD and other related cannabinoids making the results of this study specific to this industrial hemp extract that may not translate to other available products due to differing cannabinoid concentrations in this largely unregulated market.

In conclusion, this particular product was shown to be bioavailable across the small number of dogs examined in the PK portion of the study, and dogs with OA receiving this industrial hemp extract high in CBD (2 mg/kg of CBD) were perceived to be more comfortable and active. There appear to be no observed side effects of the treatment in either the dogs utilized in the PK study at 2 and 8 mg/kg, or dogs undergoing OA treatment for a month duration. There were some dogs with incidental rises in alkaline phosphatase that could be related to the treatment. Further long-term studies with larger populations are needed to identify long-term effects of CBD rich industrial hemp treatment, however short term effects appear to be positive.

## Author contributions

L-JG was responsible for data analysis and interpretation, drafting of the manuscript and approval of the submitted manuscript. JB was responsible for the conception of the study and manuscript writing and revisions. CF was responsible for acquisition of data and manuscript revision. WS was responsible for pharmacokinetic evaluation and revision of the manuscript. SM was responsible for statistical analysis, data analysis and revision of the manuscript. LW was responsible for laboratory work including liquid chromatography-mass spectrometry. HB was responsible for interpretation of the blood work and manuscript revision. EB was responsible for acquisition of data, and data analysis. JW was responsible for the conception of study, supervised data collection, statistical analysis, and manuscript editing.

### Conflict of interest statement

The authors declare that the research was conducted in the absence of any commercial or financial relationships that could be construed as a potential conflict of interest.

## Abbreviations

CBD: cannabidiol
CB: cannabinoid
CBDa: carboxylic acid of CBD
THC: tetrahydrocannabinol
CBC: cannabichromene
CBG: cannabigerol
CBPI: Canine Brief Pain Inventory.
